# An Elementary System of Physiology

**Published:** 1837-04

**Authors:** 


Art. VII.—*An Elementary System of Physiology. Complete in one Volume.* By John Bostock, m.d. f.r.s. &c. *Third Edition, revised and corrected throughout.—London.* Baldwin and Cradock. 1836. Pp. 887.

The well-established character of Dr. Bostock's Elementary System of Physiology renders it unnecessary for us to make any extended comments upon it. Since its first publication, several other treatises on the same subject have appeared in this country, all of them of great merit, and each possessing certain peculiarities of its own. Of these works, as well as of foreign systems of physiology, and of the records of various experimenters, in essays and journals, both at home and abroad, Dr. Bostock has very extensively availed himself, in this, the third edition of his work, which is now published in one volume, instead of in three volumes. Great pains have evidently been taken by the author to present an analysis of much that is valuable which has been published since the former edition; to afford the student a reference to the best authorities on the subject of his study; and to place the work on a levelwith the existing state of physiological science.

The only fault we can find with the author is, that he has (as stated in his advertisement,) "in most cases preferred appending the new matter in the form of notes to interweaving it into the text;" as we do not consider his reason for so doing, viz. "that those who are in possession of the former editions may be enabled more easily to distinguish the additions that have been made to the present," at all sufficient to make amends for the inconvenience attending the plan adopted. Had, indeed, the additions been published in a separate form, for the advantage of the possessors of the former editions, the case would have been different; but the facility of being able to *distinguish* the new from the old matter in a volume which they must purchase in order to do so, seems to us hardly worthy of consideration. The consequence of the plan adopted by the author is, that in some cases the notes are almost unconnected with, if not actually opposed to, the matter in the body of the work. Thus we observe, here and there, that an allusion only is made to the works of some authors, whose opinions merit careful consideration, and whose discoveries differ materially from the statements given in the text. In such cases it would have been much better if the text had been altered, especially where the alteration would have been probably nearer the truth than the original version of the author. An error may be thus propagated which might have been easily avoided by a little more trouble. This defect is, however, but rare; and, indeed, when it appears to be the object of the author to supply a bibliography of physiology, as well as an elementary system, it is perhaps hardly to be expected that he can have carefully estimated the value of all the books to which he refers: the deficiency is therefore one for which the nature of the subject, rather than the author, should probably bear the blame.

The profession, in requiring a third edition of Dr. Bostock's Elementary System, has acknowledged the merits of those editions which preceded it; merits which will be in no degree diminished by the character of the volume which is now before us. In point of extent and completeness, indeed, this work must take precedence of any other in our language: and if, when another edition is called for, as will no doubt be the case ere long, the author will adopt our suggestion of amalgamating the new matter with the old, in the text, throughout, it will be difficult to find its equal in this or any other language.

